# Amlodipine-induced buccal lichenoid lesions: A case report

**DOI:** 10.51866/cr.531

**Published:** 2024-03-22

**Authors:** Mohamed Pauzi Muhammad Hafiz, Abdul Kadir Azidah, Mat Yudin Zainab

**Affiliations:** 1 MD, MMed, PhD, Department of Family Medicine, School of Medical Sciences, Health Campus, Universiti Sains Malaysia, Kubang Kerian, Kelantan, Malaysia. Email: azidahkb@usm.my; 2 MBBCh, Department of Family Medicine, Universiti Sains Malaysia, Kubang Kerian, Kelantan, Malaysia.; 3 MD, MMed Fam Med, School of Dental Sciences, Universiti Sains Malaysia, Kubang Kerian, Kelantan, Malaysia.

**Keywords:** Amlodipine, Adverse drug reaction, Lichenoid eruptions, Lichen planus, Oral ulcer

## Abstract

Buccal lichenoid lesions (BLLs) are characterised by a unique, linear whitish striation in the buccal region and can be accompanied by ulcers, plaques, erythemas, atrophies and blisters. They are distinguished from oral lichen planus (OLP) by the association of the administration of a drug or contact with a metal. Herein, we present the case of a 42-year-old woman with underlying hypertension with amlodipine-induced BLLs. She complained of a 1-month history of right buccal whitish streaks and oral ulcers 2 months after taking amlodipine. She visited a private otorhinolaryngology clinic, and a biopsy for the right buccal ulcer was conducted. The biopsy result showed features suggestive of OLP. The patient was then diagnosed with OLP. Her symptoms were persistent despite treatment, so a dental referral was made. Amlodipine was suspected as the cause of her condition and was therefore stopped. Her condition gradually resolved after amlodipine withdrawal. Hence, primary care physicians should be aware of BLLs as one of the adverse drug reactions of amlodipine so that prompt management can be taken to avoid further debilitating impacts on patients.

## Introduction

A buccal lichenoid lesion (BLL) is a type of oral lichenoid disease (OLD) described as a linear, reticular, whitish papule in the buccal mucosa. Other sites can be the tongue and gums. OLD can also present with painful ulceration, erosion, erythema, atrophic change or plaque or bullous formation.^1^ The prevalence of OLD is higher among women aged 55–57 years than among men.1 In the United States, the incidence of a serious drug reaction ranges from 2.4% to 16.2%, and such a reaction often presents with oral manifestations. Amlodipine-induced oral hypersensitivity reactions account for less than 1% of these cases.^2^ A BLL shares similar clinical and histopathological presentations with oral lichen planus (OLP), which is a chronic autoimmune disorder, making both challenging to differentiate from each other. A history of exposure to dental materials such as palladium and amalgam or administration of drugs such as non-steroidal anti-inflammatory drugs, allopurinol, amlodipine, beta-blockers, topical glucocorticoids and rituximab before the lesion occurrence is the distinctive feature of a BLL.^1-4^ In the medical literature, there have been only few reported cases of amlodipine-induced BLLs.^5^ Herein, we present a case of amlodipine-induced BLLs, initially treated as OLP, which was re-evaluated and correctly diagnosed as being induced by amlodipine. This change in diagnosis was prompted by the realisation that the patient had started taking amlodipine before the appearance of the lesions.

## Case presentation

A 42-year-old Chinese woman with a known case of newly diagnosed hypertension presented with progressively worsening right buccal whitish streaks and multiple painful ulcers for 1 month. She denied fever, lethargy, joint pain and other symptoms. The patient had a history of 1-day hospitalisation in a private hospital for hypertensive urgency and was started on oral amlodipine 5 mg OD 2 months prior to the onset of her symptoms. She was given a follow-up date but defaulted due to her busy work schedule.

Initially, the patient visited a private otorhinolaryngology clinic, as the buccal ulcers became excruciatingly painful with a pain score of 8 out of 10. The pain had severely affected her routine activities. A painkiller was given, and her pain was slightly reduced. She denied any history of oral ulcers and family history of oral cancer or autoimmune diseases. Infective screening for human immunodeficiency virus infection, hepatitis and syphilis revealed negative results. A biopsy for the right buccal ulcer was conducted, and the result revealed a few pieces of inflamed and ulcerated mucosal tissues consisting of epithelium and subepithelial stroma. There was a band-like lymphohistiocytic infiltrate in the subepithelial stroma associated with vacuolar degenerative basal cell changes and the presence of Civatte bodies. A few squamous epithelial nests in the inflamed stroma and scattered melanophages were present. The epithelium and squamous epithelial nests displayed vesicular nuclei, prominent nucleoli and ample pale eosinophilic cytoplasm with prominent intercellular bridges eliciting reactive atypia. No obvious dysplasia or malignancy was observed. The patient was diagnosed with OLP, and amlodipine was continued for her hypertension. She was subsequently referred to the otorhinolaryngology department within our health centre due to her relocation to a new work placement.

**Figure 1:**
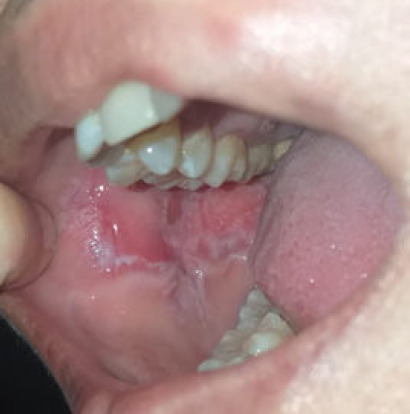
Right buccal whitish papules with erythematous ill-defined margin ulcers extending to the posterior gingival sulcus due to amlodipine administration.

Oral examination revealed an inflamed buccal region with whitish streaks and multiple ulcers extending to the posterior gingival sulcus (Figure 1). No cervical lymph nodes were palpable. Other oral structures and systemic examination findings were normal. Her full blood count, renal function and liver function were also normal. She was treated for OLP with infected oral ulcers, and a 1-week course of oral amoxicillin/clavulanic acid 625 mg BD treatment was commenced. During her follow-up at 2 months, she still complained of persistent painful right buccal ulcerative whitish streaks. The managing team then started her on a 6-week course of local clobetasol propionate treatment and a 2-week course of oral prednisolone 60 mg OD treatment. A dental referral was made for further expert evaluation.

**Figure 2:**
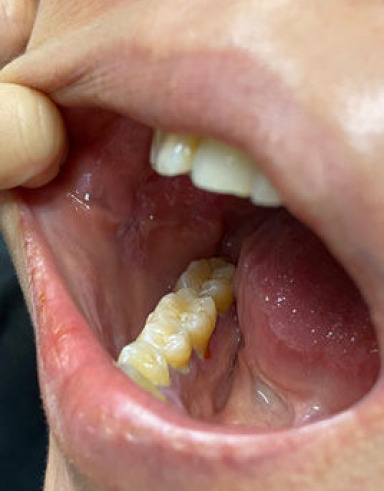
Minimal whitish papules in the right buccal region with complete resolution of ulcers after amlodipine discontinuation.

Upon review by the dental team, there was a suspicion that the whitish buccal lesions might be a lichenoid reaction caused by amlodipine. Thus, amlodipine was withdrawn, and the patient was referred to our primary care clinic for appropriate antihypertensive selection and blood pressure monitoring. During our review, her blood pressure was 122/91 mmHg, and her pulse rate was 96 beats/min. We counselled her on oral perindopril 4 mg OD as a replacement for oral amlodipine, and a subsequent follow-up date was given. Four weeks after amlodipine withdrawal, she reported that her symptoms were markedly alleviated and that she had not experienced any pain. On oral examination, there were minimal whitish streaks over the right buccal region with complete resolution of ulcers (Figure 2). Her home blood pressure monitoring chart recorded uncontrolled diastolic pressure ranging from 91 to 96 mmHg. Upon further questioning regarding adherence, she admitted that she had not taken a single tablet of perindopril since prescribed due to fear of medication side effects. She was not informed earlier that the occurrence of whitish lesions and painful ulcers in the mouth is among the potential side effects of amlodipine before starting it. Following an open discussion addressing her concerns, the patient ultimately consented to begin taking perindopril to manage her hypertension with scheduled follow-ups. Regarding her anxiety, psychoeducation was provided to help her comprehend her medical problem and alleviate her worries.

## Discussion

Amlodipine is a calcium-channel blocker and the second-line treatment for hypertension. The common side effects of amlodipine are headache, dizziness, peripheral oedema, flushing and fatigue. A BLL is a rare adverse drug reaction of amlodipine.

The exact mechanisms behind the development of a BLL due to medications such as amlodipine are unknown. It is believed that they involve a cell-mediated immunologic response similar to that seen in OLE^6,7^ The susceptibility to adverse drug reactions is determined by genetic factors and immune dysregulation.^7^ To date, only one case of amlodipine-induced lichenoid drug eruption has been reported in Malaysia.^5^ The patient had multiple lesions mainly on the tongue and right buccal mucosa, followed by violaceous papules on the right leg, in contrast to our case.

A BLL is a clinicopathological diagnosis. It presents as a whitish papule and is distributed in linear and reticular patterns.^1^ It can appear as ulcerative, erosive, atrophic or bullous in the buccal or oral mucosal regions.^1,3^ A BLL is usually unilateral and asymmetrical, whereas OLP commonly appears as bilateral and symmetrical.^1,3^

Histopathological examination is essential to confirm the diagnosis of a BLL. It can reveal specific characteristics such as inflammatory cell infiltration, basal cell liquefaction degeneration and saw-tooth rete ridges. Additionally, the presence of colloid bodies, Civatte bodies and apoptotic keratinocytes can be observed.^1,3,6^ These findings aid in the diagnosis and differentiation of a BLL from other oral conditions.

A BLL carries a potential risk of malignant transformation.^1^ This is because prolonged inflammation can damage DNA, cause mutations in cells and create a microenvironment conducive to the development of cancerous cells. A malignancy rate of 2.6% was demonstrated in a previous cohort study involving 384 patients with OLD.^1^ Further, the malignancy rate for OLP and oral lichenoid lesions was 1.7% and 5.9%, respectively.

The definitive management of a BLL is the removal of causative medications, resolving symptoms.^1^ However, notably, the resolution of symptoms might not be immediate, as the inflammatory process triggered by medications may need time to subside. In our case, the patient developed right buccal ulcerative whitish papules after 2 months of amlodipine initiation, and these symptoms persisted for 19 months while she continued to take the medication. After suspicion of amlodipine as the cause, the medication was discontinued, resulting in a gradual alleviation of the symptoms.

A BLL can be misdiagnosed as OLP, as both have similar clinical and histopathological appearances, but a BLL has a distinguishable feature that can help differentiate it from OLP. The key feature is a history of exposure to potential medications or allergens before the onset of the lesion. Our patient was misdiagnosed to have OLP at her initial presentation, and inappropriate management had caused the unresolved symptoms. Therefore, it is crucial for primary care physicians to recognise the clinical features of a BLL and obtain an extensive medication history from patients to accurately diagnose it so that further complications can be avoided, and correct treatment can be given promptly.

## Conclusion

A BLL is a rare side effect of amlodipine. The presented case highlights the importance of timely identification of this drug-related lesion, which is characterised by unique, linear whitish papules with painful oral ulcers. It also underscores the significance of considering medication-related aetiologies of oral mucosal changes. With discontinuation of amlodipine, a gradual improvement in symptoms is observed. Therefore, vigilant monitoring for adverse drug reactions in patients taking amlodipine should be practised.
